# Variations in the anatomical relationship between the common carotid artery and the internal jugular vein: An ultrasonographic study

**Published:** 2015-06-30

**Authors:** Mauricio Umaña, Alberto García, Luis Bustamante, José Luis Castillo, Juan Sebastián Martínez

**Affiliations:** 1 Emergency Physician. Intensive Care Fellow, Universidad del Valle, Fundación Valle del Lili, Cali, Valle, Colombia; 2 Surgeon Intensivist. Universidad del Valle, Intensive Care Unit, Fundación Valle del Lili, Cali, Valle, Colombia; 3 General Surgeon. Intensive Care Fellow, Universidad del Valle, Fundación Valle del Lili, Cali, Valle, Colombia

**Keywords:** Ultrasound, carotid artery, internal jugular vein, central venous catheterization, anatomy

## Abstract

**Introduction:**

The internal jugular vein locates anterior or anterolateral to the common carotid artery in two-thirds of the subjects studied by ultrasound when the head is in a rotated position.

**Aim:**

To identify variables associated with the anterior location of the internal jugular vein.

**Methods:**

Ultrasound examinations were performed with the patients in the supine position, with the head rotated to the opposite side. The proximal third of the neck was visualized transversely with a 7.5-mHz transducer. The relationship between the vessels was described in accordance with the proportion of the artery overlapped by the vein. Univariate comparisons and a multivariate analysis of potential variables that may affect the anatomic relationships were performed.

**Results:**

Seventy-eight patients were included, 44 of whom were men. The patients' ages ranged from 17 to 90 years (median 64.0, interquartile range 41-73). The right and left sides were studied 75 and 73 times, respectively. The vein was located lateral to the artery in 24.3% (95%CI= 17.4-32.2) of the studies, anterolateral in 33.8% (95%CI= 26.2-41.4) and anterior in 41.9% (95%CI= 33.9-49.8). The multivariate analysis identified age group (OR= 3.7, 95% CI 2.1-6.4) and, less significantly, the left side (OR= 1.7, 95%CI= 0.8-3.5) and male gender (OR= 1.2, 95%CI= 0.6-2.7) as variables associated with the anterior position of the vein.

**Conclusión:**

The anterior position of the internal jugular vein relative to the common carotid artery increases gradually with age. Additionally, left-sided localization and male sex further increased the probability of an anterior position.

## Abstract (es)

**Introducción:**

La vena yugular interna es anterior o anterolateral a la arteria carótida común en las dos terceras partes de los sujetos estudiados sonográficamente, con la cabeza rotada.

**Objetivo:**

Se examinó la asociación de diferentes variables con la ubicación anterior de la vena.

**Métodos:**

Las ecografías se realizaron en posición supino, con la cabeza rotada hacia el lado contrario al examinado. Se visualizó transversalmente el tercio proximal del cuello, con un transductor de 7.5 mHz. La relación entre los vasos se describió de acuerdo con la proporción de la arteria cubierta por la vena. Se hicieron comparaciones univariadas con la prueba Chi^2^ de Pearson y un análisis multivariado de las variables candidatas a afectar las relaciones anatómicas estudiadas

**Resultados:**

Se incluyeron 78 individuos, 44 hombres, con edad entre 17-90 años (mediana 64.0, rango= 41-73 años). Se estudió el lado derecho en 75 ocasiones y el izquierdo en 73. La vena se localizó lateral en el 24.3% (IC95% 17.4-32.2) de los vasos estudiados, anterolateral en el 33.8% (IC95%= 26.2-41.4) y anterior en el 41.9% (IC95%= 33.9-49.8). El análisis multivariado identificó: el grupo etáreo (OR= 3.7, IC95%= 2.1-6.4) y sugiere el lado izquierdo (OR= 1.7, IC95%= 0.8-3.5) y el género masculino (OR= 1.2, IC95%= 0.6-2.7), como variables asociadas con la posición anterior de la vena.

**Conclusion:**

La ubicación anterior de la vena yugular interna respecto a la arteria carótida común aumenta gradualmente con la edad. La localización izquierda y el género masculino pueden aumentar adicionalmente esta probabilidad.

## Introduction

The cannulation of central venous accesses is a widely used procedure in medical practice. This procedure is used for hemodynamic monitoring, the administration of fluids, the delivery of irritating or hyperosmolar drugs, the supply of parenteral nutrition, and for hemodialysis 1-3, among other purposes.

Although the internal jugular vein is a superficial and easily accessible structure, complications related to catheter insertion are common (10-11%) 3,4 In some instances, such as in cases of the obstruction of the airway by an expanding hematoma, arterial puncture, pleural puncture and secondary pneumothorax, these complications can threaten the patient's life. Real-time ultrasound imaging has been very useful for locating the vessel to guide the puncture. Although this method of visualization aids in vessel location, the possibility of complications remains 2,5,6.

The ultrasonographic study of the relationships between the internal jugular vein and the carotid artery results in variable outcomes. These outcomes depend partially on the imaging technique employed, the definitions utilized and racial issues that have not yet been clarified 7-10. Different researchers have noted than an anterior position of the vein that partially or completely overlies the artery is a risk factor for arterial puncture during venous cannulation 9,11,12. The proportion of subjects in whom the artery overlaps the vein to a significant can fluctuate between 15% and 54% 8,9. The vein may completely cover the artery in 10% of cases or may partially cover the artery in 57% of cases 13. It has been suggested that this condition is exacerbated with the rotation of the head, in men, and on the right side and that the condition increases with age 14-17. In one-third of cases in our experience, the vein moves medially with the rotation of the head; the vein moves laterally in one-third of cases and does not change in position in the remaining third. After head rotation, 11% of the veins completely covered the artery 13.

In this study, the association between sex, age and assessed side and the anatomical relationships between the internal jugular vein and the common carotid artery were observed by ultrasound. These results could be useful in cases of ultrasound-guided insertions as well as in in those guided by anatomical repair.

## Materials and Methods

A cross-sectional study was conducted to describe the anatomical relationships of the internal jugular vein and the carotid artery in the base of the neck, and the characteristics that predisposed to an anterior position of the vein overlaying the artery were also examined by ultrasound. Seventy eight inpatients from a mixed intensive care unit (ICU) were included in the study. The patients were hemodynamically stable and attended consecutively. Patients with any of the following characteristics were excluded: anatomic abnormalities that may distort vascular relationships, hospitalization for surgical procedures or a traumatic disease or tumor in the neck, and catheter placed in the internal jugular vein.

### Sample size

The required sample size to compare two paired samples and obtain a 20%-35% difference in proportions with a 95% confidence interval and a power of 80% was calculated. It was estimated that the required sample size was 151 patients. Sample size and statistical analysis were performed using the statistical package Stata^®^ 12.1 (Mac, Corp College Station, TX, USA). 

### Ultrasound technique and interpretation

The examination technique was standardized, and all of the examinations were implemented by some of the researchers, who previously received training and certification for the practice of ultrasound-guided internal vascular access 14. On 20 occasions, the study was interpreted by two independent researchers who were blind to the interpretation of the other examiner. In each of these studies, both researchers agreed on the interpretation of the anatomical relationships. 

**Figure 1.**
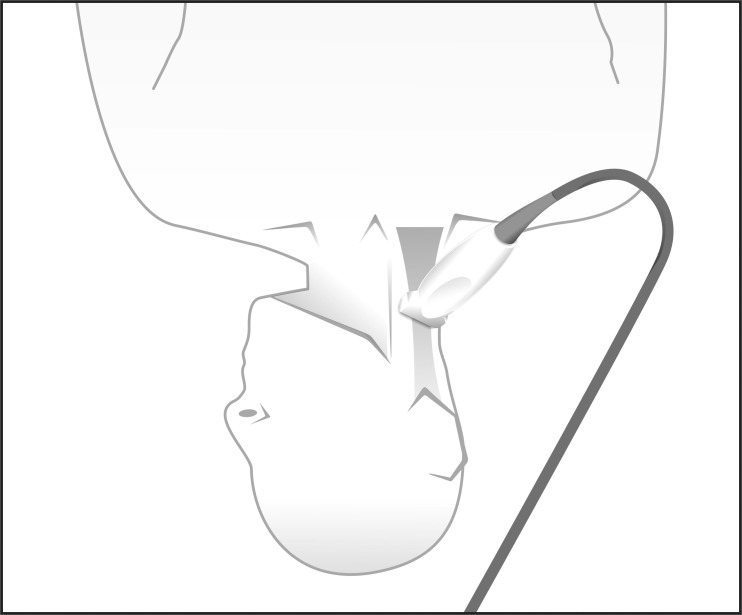
Patient positioning for the examination. The head is rotated to the opposite side and the probe is oriented transversely, perpendicular to the axis of the vessels.

**Figure 2.**
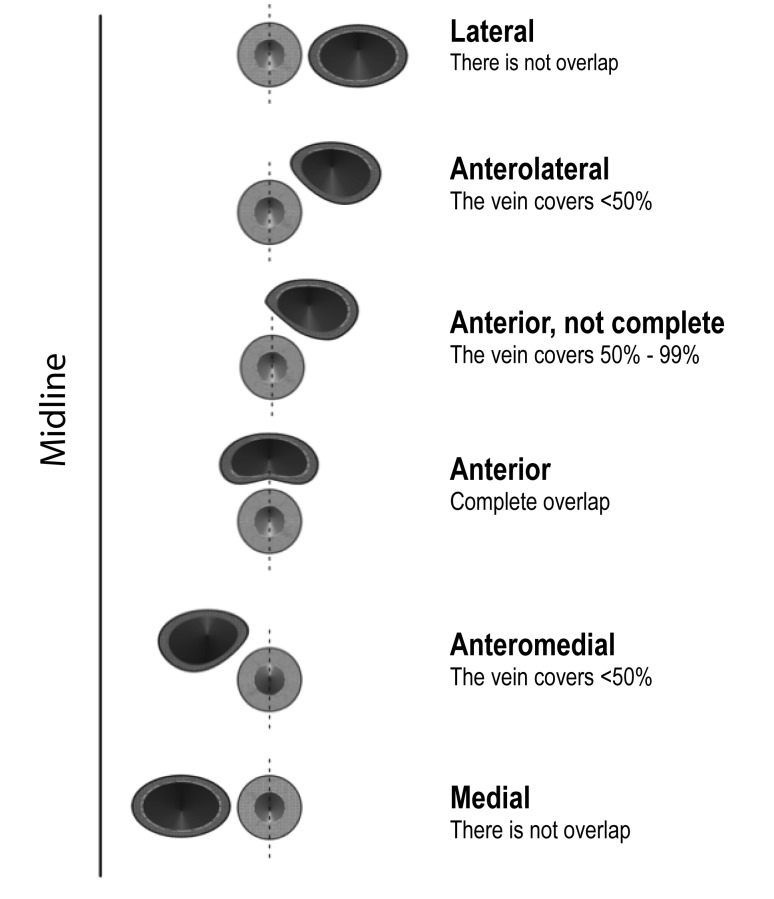
Classification of the anatomical relationship between the internal jugular vein and common carotid artery.

The ultrasound study was performed with the patient in the supine position. The head was initially in a neutral position and was then rotated toward the side opposite of the side to be assessed. The proximal third of the neck was visualized transversely with a Sonosite 180 or Sonosite EDGE (Sonosite, Bothell, Washington) ultrasound using a 7.5-mHz linear transducer. The reference point of the device was directed to the midline. (Figs. 1  and 2). The data were digitally saved for later analysis. 

**Figure 3.**
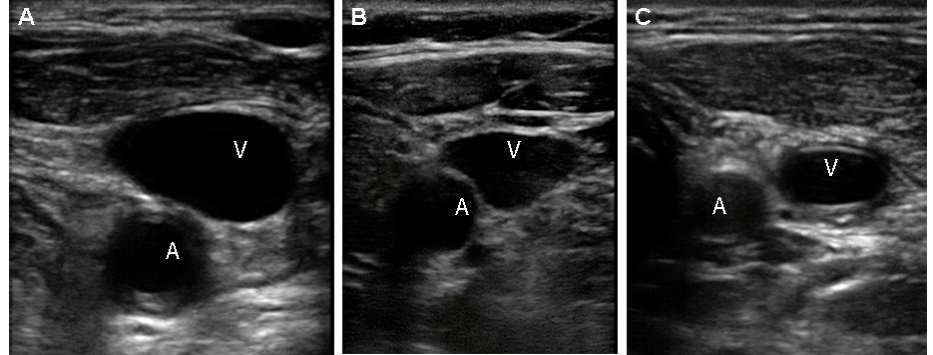
Examples of different magnitudes of overlap. A. Anterior: The vein covers completely the artery. B. Antero-lateral: The vein covers to 50% of the artery. C. Lateral: The vein is located lateral to the artery.

 The relationship between the vessels was described according to the proportion of the artery overlapped by the vein (Fig. 3), consistent with the results reported by other authors 9,15,18-22. This method of describing the relationship between vascular structures was chosen instead of those describing the proportion of overlap in degrees 10,17 or the magnitude of overlap or separation 23. 

The influence of several variables on the relationship between the vein and the artery was analyzed with data obtained from the studies performed with the head in the rotated position. The relationship was dichotomized as "non-significant overlap" and "significant overlap". "Non-significant overlap" was diagnosed when the vein was in a completely lateral position or in an anterolateral position to the artery without covering 50% of the arterial lumen. "Significant overlap" was diagnosed when the vein covered more than 50% of the artery or was in an anteromedial or medial position. The differences depending on the left or right position, age group and gender were analyzed. The patient's data and relationships in different positions were recorded in a precoded format. 

### Statistical analysis

Continuous variables are reported as range, median and interquartile range. Discrete variables are reported as quantities and proportions. The proportions were compared using Pearson's Chi-square (Chi^2^) test. A multivariate logistic regression model was constructed to determine the contribution of each variable to the location of the vein in a position of "significant overlap".

### Ethical considerations

This research was considered of minimal risk according to Resolution 8430 of the Colombian Ministry of Health24. Information was collected and stored during the routine patient exam, with the patient's consent, to be used as teaching material. The demographic data were handled anonymously. No sensitive information was recorded. The protocol was approved by the Ethics Committee of Fundación Valle del Lili (approval 046-213). 

## Results

Seventy eight patients, 44 (56.4%) of whom were men, were included in the study (Table 1). The patient ages ranged from 17 to 90 yrs with a median of 64 yrs and an interquartile range of 41-73.

**Table 1.**
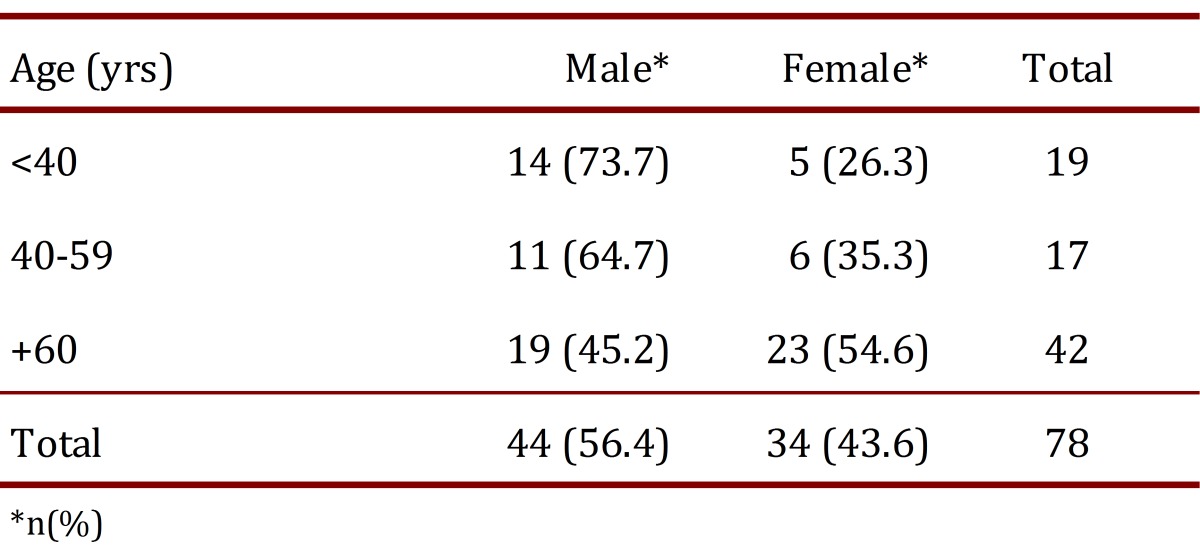
Age and gender distribution of patients included in the ultrasonographic study.

A total of 148 studies were collected between October 2012 and March 2013. Seventy five (51%) of the studies involved the right side, and 73 (49%) involved the left side. On eight occasions, only one side was examined due to limitations during rotation or to the presence of a catheter. Two patients were excluded because images were damaged during the storage process.

In the neutral position, the internal jugular vein significantly overlapped the common carotid artery in 23 out of 75 (30.7%) observations on the right side and 30 out of 73 (41.1%) observations on the left side (Table 2). When the head was rotated to the opposite side, significant overlap was observed in 28 out of 75 (37.3%) observations on the right side and in 34 out of 73 (46.6%) observations on the left side (Table 2).

**Table 2.**
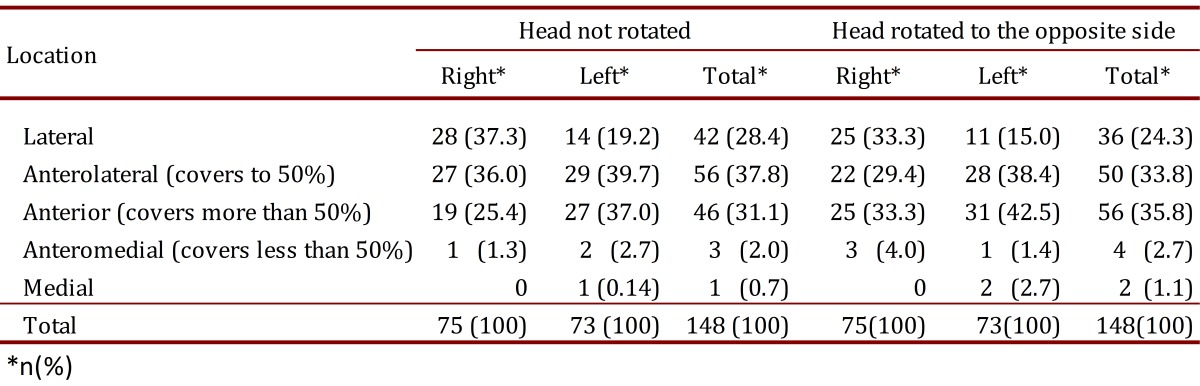
Anatomical relationship of the common carotid artery and the internal jugular vein, according to the assessed side and the position of the head, rotated or not.

Analysis of factors associated with an overlap of least half of the common carotid artery by the internal jugular vein

The proportion of vessels with significant overlap of the artery was 35.4% in males and 50.0% in females (OR= 1.83; 95%CI= 0.89-3.74; *p*= 0.07). This finding was more common on the left side, 46.6% vs. 37.3% (OR= 1.46; 95%CI= 0.72-2.97; *p*= 0.25), and increased significantly with increasing age (Table 3). The proportion of men and women differed according to the age group. In the group of patients younger than 40 yrs, the men:women ratio was 3:1, whereas in patients aged 40-59 yrs, the ratio was 2:1, and in patients aged 60 yrs or more, the ratio was close to 1:1 (Table 1).

**Table 3.**
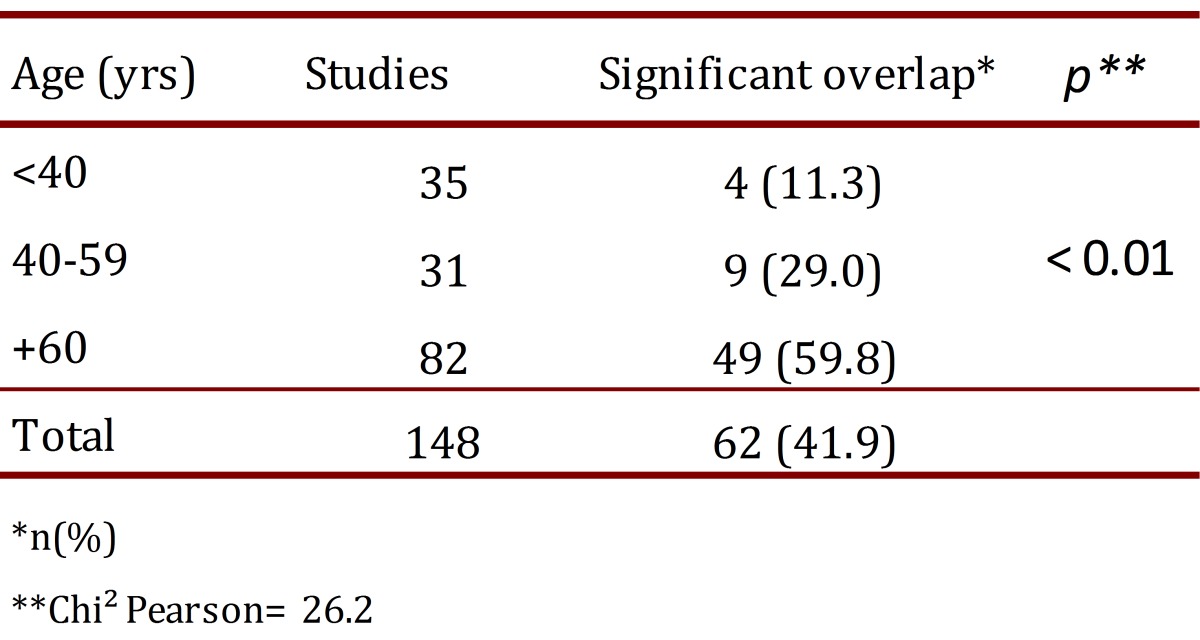
Significant overlap of the common carotid artery, according to age groups.

The results of the multivariate analysis are presented in Table 4. The results confirm an association between older age and a higher probability of significant overlap of the artery. The adjustment of the multivariate analysis rejects the apparent protective effect of being a male and confirms a strong confounding effect determined by the unequal gender distribution within the age groups.

**Table 4.**
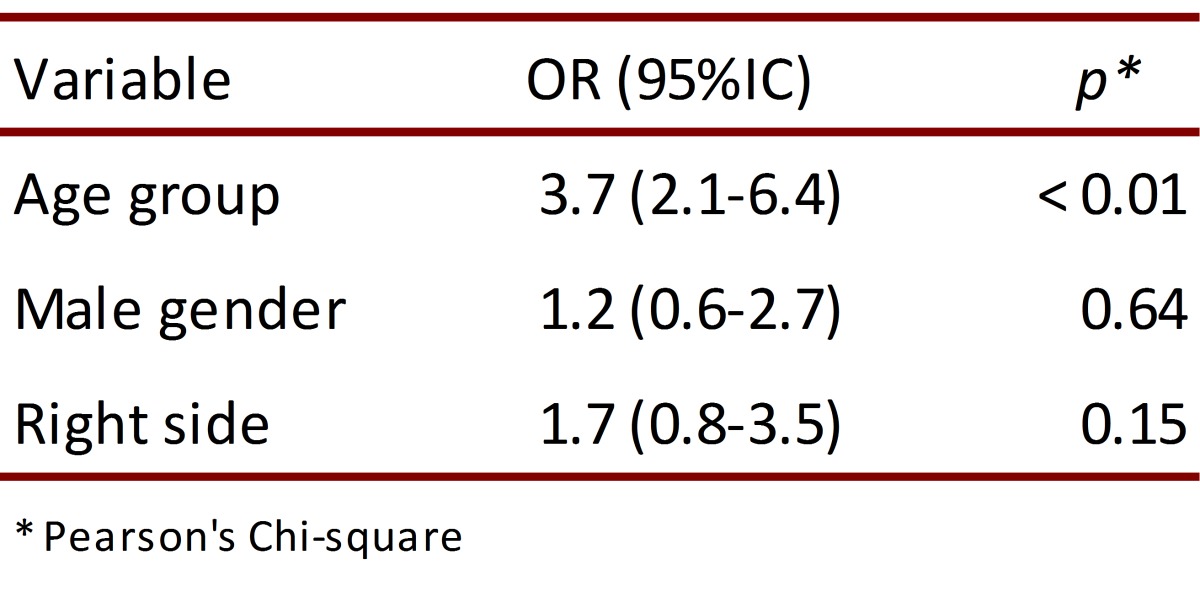
Multivariate analysis of the variables associated with the significant overlap of the carotid artery.

## Discussion

Venous vascular access via the internal jugular is a common practice in healthcare services with different objectives. During its implementation, it is common that the operator rotates the patient's head to the opposite side. According to our results, this movement might cause the internal jugular vein to cover the common carotid artery, increasing the probability of an inconspicuous puncture of the carotid artery, which could trigger the development of an expansive hematoma, an arteriovenous fistula or a mistake in a venous device implantation, leading to their respective consequences. In our case, this overlap was less frequent than that published by other authors 17,25-27, and in our patients, the overlap occurred in the medial position in some cases and in the lateral position in others. These differences with regard to other the findings of other authors could be explained by the particular features of our population, to the age distribution of the different groups of patients reported by other authors, and by the ultrasound technique itself, as demonstrated by Sibai *et al*., who observed a higher proportion of artery overlap when the transducer was oriented parallel to the skin of the neck and not vertically 11.

Shoja *et al*. reported a higher predisposition to artery overlap in men 28. Interestingly, our univariate analysis identified an apparent lower probability of significant overlap of the artery in men. The analysis of this phenomenon in the different age groups revealed that the high proportion of men in the groups under sixty, who are less likely to exhibit this overlap, explains this finding. The adjustment in the multivariate test supported this confounding effect and suggests that male sex may represent a variable that can be associated with an increased risk of overlap.

Previous research results are controversial with respect to the predisposition of one side or another to a greater likelihood of overlap 16,27-29. However, there is an obvious asymmetry 30 and greater difficulty in performing the cannulation of the vein on the left side, which is associated with a greater probability of complications 31,32. Our data suggest a greater predisposition to significant overlap on the left side, but we are unable to confirm or rule out this predisposition due to insufficient power for a difference in the order of that observed in this variable.

The most important result of our research is the confirmation of the association between advanced age and predisposition of the internal jugular vein to overlap the common carotid through a multivariate analysis. This finding was suggested by Troianos *et al*., in a sample of 1,136 patients undergoing elective surgery 9.

The potential limitations of the study were that a random sampling system that fully guarantees the representativeness of the sample was not used. However, the patients were recruited from all divisions of the unit in which the research took place. A unified system to rank the relationships between the internal jugular vein and the carotid artery does not exist in the literature. Furthermore, the technique of ultrasound exploration is not standardized. In our case, the ultrasound technique was standardized, and a published classification system of the relationships 9 was adapted. The sample size is insufficient to confirm or reject the finding of a lack of significant associations that involve gender and side.

## Conclusions

The probability that the internal jugular vein significantly covers the common carotid artery in the lower neck increases gradually with age. To study the effects of other proposed associations, a research study with the suitable power must be performed.
